# MRI-Based Deep Learning Image Classification for Screening Temporomandibular Joint Degenerative Joint Disease

**DOI:** 10.3390/jcm15166225

**Published:** 2026-08-11

**Authors:** Liang Xu, Kaixi Qiu, Weiliang Wu, Xiaofeng Zhu, Jiang Chen

**Affiliations:** 1 School of Stomatology, Fujian Medical University, Fuzhou 350000, China; dentistxl1987@163.com (L.X.); wuweiliang1981@vip.163.com (W.W.); 2 Department of Stomatology, The First Affiliated Hospital of Fujian Medical University, Fuzhou 350005, China; dentzxf@163.com; 3 Department of Stomatology, National Regional Medical Center, Binhai Campus of the First Affiliated Hospital, Fujian Medical University, Fuzhou 350212, China; 4 Department of Stomatology, Fuzhou General Hospital Affiliated with Fujian Medical University, Fuzhou 350005, China; qkxdentist@163.com; 5 School and Hospital of Stomatology, Fujian Medical University, Fuzhou 350000, China

**Keywords:** deep learning, temporomandibular joint degenerative joint disease, magnetic resonance imaging, automatic classification

## Abstract

**Background:** This study aimed to develop and evaluate a deep learning (DL) algorithm based on magnetic resonance imaging (MRI) for the classification of temporomandibular joint degenerative joint disease (TMJDJD), thereby exploring its potential role in assisting cone-beam computed tomography (CBCT) examinations and minimizing patient radiation exposure. **Methods:** A retrospective analysis was conducted on 104 patients who had undergone both MRI and CBCT examinations of the temporomandibular joint. A total of 1769 sagittal and 1448 coronal MRI images were collected. After image preprocessing, the datasets were grouped according to different imaging features. Three DL frameworks—ResNet101, DenseNet201, and MobileNetV2—were developed to classify TMJDJD using various MRI image categories. The classification performance of these models was evaluated using accuracy, precision, recall, F1 score, Matthews correlation coefficient (MCC), and the results were compared with classifications made by two senior clinical experts in a blinded image-level reader experiment. **Results:** DenseNet201 achieved the best performance in the sagittal (Sg) group, with an accuracy of 80.11%, precision of 80.26%, recall of 75.31%, and an MCC of 0.60. DenseNet201 achieved the best performance among the evaluated DL models, with higher overall performance metrics than ResNet101 and MobileNetV2. In a constrained image-level reader comparison, DenseNet201 demonstrated comparable overall agreement metrics to the two experts, although one expert achieved higher recall and a lower false-negative rate. t-Distributed Stochastic Neighbor Embedding (t-SNE) and training curve analyses confirmed that DenseNet201 demonstrated superior feature extraction capability and a more stable training process. Grouping MRI images, however, did not improve model accuracy. **Conclusions:** DenseNet201 showed preliminary potential for MRI-based TMJDJD classification, particularly on sagittal images. Based on image-level classification results, the model may provide preliminary support for identifying MRI findings associated with TMJDJD and assisting decisions regarding further CBCT evaluation.

## 1. Introduction

Patients with temporomandibular joint disorder (TMD) often require cone-beam computed tomography (CBCT) to diagnose degenerative joint diseases, while magnetic resonance imaging (MRI) is also necessary to confirm joint disc displacement. CBCT has the advantage of visualizing bone structures and can clearly detect pathological changes in the condyle, such as bone resorption, sclerosis, and cystic degeneration [1]. In contrast, MRI provides detailed imaging of the temporomandibular joint (TMJ) and surrounding soft tissues, which is essential for evaluating structures such as the articular disc and joint capsule [2]. It is well established that a single imaging modality often fails to capture the complete pathological profile of TMD. Therefore, integrating MRI and CBCT data has become standard practice to achieve a more comprehensive diagnostic evaluation. However, this diagnostic approach presents two major challenges for clinicians. Firstly, although the radiation dose of CBCT is lower than that of conventional computed tomography (CT), with effective doses typically ranging from 10–670 μSv [3], cumulative radiation in the head and neck region may still pose potential risks to sensitive tissues such as the eyes, thyroid gland, and salivary glands [4]. Because patients may undergo multiple imaging examinations, cumulative radiation exposure over time could increase the risk of cancer. Therefore, performing CBCT for all patients would lead to unnecessary radiation exposure. For those without bone-related lesions, this constitutes overtreatment. Secondly, CBCT has inherent limitations in diagnostic capability, as it cannot visualize soft tissues. In contrast, MRI clearly visualizes the articular disc and surrounding soft tissues while retaining substantial diagnostic value for bone structures [5,6]. Therefore, MRI serves as an important imaging modality for patients with suspected soft tissue abnormalities or internal derangement. Ideally, the diagnostic process should begin with MRI to evaluate the articular disc and condyle. For patients with a high clinical suspicion of temporomandibular joint degenerative joint disease (TMJDJD), clinicians can then decide whether supplementary CBCT imaging is necessary. This approach aligns with evidence-based medicine and medical ethics principles, minimizing radiation exposure while ensuring diagnostic accuracy.

In recent years, the rapid advancement of artificial intelligence (AI) has driven significant progress in deep learning (DL) for joint-related research, particularly in the field of medical image analysis [7]. For example, Abdullah et al. developed an AI-based tool to locate the knee joint on X-ray images and assess the severity of knee osteoarthritis according to grading standards, thereby reducing the substantial time required for subjective evaluation and diagnosis [8]. Similarly, Sekiya et al. investigated AI-assisted three-dimensional MRI analysis to evaluate early knee osteoarthritis, examining the relationships among Kellgren–Lawrence grading, medial meniscus extrusion, and cartilage thickness [9]. These notable developments have strengthened medical image analysis research and introduced a new paradigm for advancing the differential diagnosis of TMJDJD. Building upon the success of these algorithms, DL technology can be effectively applied to MRI image analysis for automated classification of TMJDJD-related osseous changes. This application offers actionable clinical insights for healthcare professionals, improving diagnostic efficiency and supporting more effective patient care.

In this study, we developed three DL models capable of extracting imaging features of the condyle from MRI scans to enable automated classification of TMJDJD-related changes. Through model validation and performance comparison using identical training datasets, we selected a DL model demonstrating superior feasibility and practicality. This model can function as a rapid and relatively accurate screening tool to assist dentists in evaluating whether further CBCT examination may be warranted, thereby improving the efficiency of preliminary TMJDJD assessment. Additionally, the model can support radiologists in diagnostic interpretation. Overall, this study offers new insights and directions for the application of DL in medical image analysis, fostering the broader adoption and advancement of artificial intelligence in healthcare.

## 2. Methods and Materials

### 2.1. Study Population and Data Collection

This study included patients presenting with TMJ-related symptoms at the Oral Medicine Center of the First Affiliated Hospital of Fujian Medical University between 2020 and 2024. All patients underwent both MRI and CBCT of the TMJ region as part of their clinical evaluation. CBCT images were obtained by the Radiology Department using a KaVo i-CAT system (KaVo Group, Los Angeles, CA, USA) under the following parameters: X-ray tube voltage of 120 kV, current of 5 mA, exposure time of 4 s, and resolution of 0.25 mm. MRI images were acquired using a Magnetom Prisma scanner (Siemens Healthineers, Munich, Germany) with the following parameters: field strength of 3.0 Tesla, magnet bore diameter of 60 cm, magnet length of 213 cm, slice thickness of 2.2 mm, field of view of 14 × 14 cm, and gradient field strength of ≥80 mT/m at 200 T/m/s. The MRI protocol included proton density–weighted and T1-weighted sequences. All MRI scans were performed in both open-mouth and closed-mouth positions. For each subject, sagittal and coronal images were obtained, covering both mouth positions.

The inclusion and exclusion criteria for this study were defined as follows:

Inclusion Criteria:

- No history of oral or maxillofacial trauma or surgery;
- No evident facial asymmetry or deviation;
- No malocclusion, such as mandibular overgrowth or retrusion;
- No TMJ ankylosis;
- No TMJ-related treatment within the previous year.

Exclusion Criteria:

- Patients with abnormal head positioning during imaging (e.g., head tilted upward or downward);
- Patients with unclear images or imaging artifacts;
- Patients lacking CBCT or MRI scans obtained within the previous month.

Two senior radiologists independently evaluated all cases according to the inclusion and exclusion criteria. Any discrepancies were resolved through discussion to ensure adherence to the study requirements. In total, 208 joints from 104 patients were included in the analysis. The mean age of participants was 24.9 years, with a median age of 22 years (IQR: 19.0–26.7 years; range: 13–75 years), comprising 25 males and 79 females.

### 2.2. CBCT Reference Standard and Joint-Level Labeling

CBCT served as the reference standard for determining the presence or absence of TMJDJD at the joint level. Two senior oral and maxillofacial radiologists independently reviewed the CBCT images of each temporomandibular joint while blinded to the MRI images and model outputs. A joint was labeled as TMJDJD if at least one of the following osseous findings was present: subchondral cyst, erosion, diffuse sclerosis, or osteophyte. Joints without these osseous findings were labeled as non-degenerative. Disagreements between the two readers were resolved by consensus; when consensus could not be reached, a third senior radiologist adjudicated the final label. The final CBCT-based joint-level label was then assigned to all MRI slices corresponding to the same joint for model training and evaluation. Interobserver agreement between the two initial readers was assessed using Cohen’s kappa at the joint level.

### 2.3. Image Preprocessing

**Figure 1:**
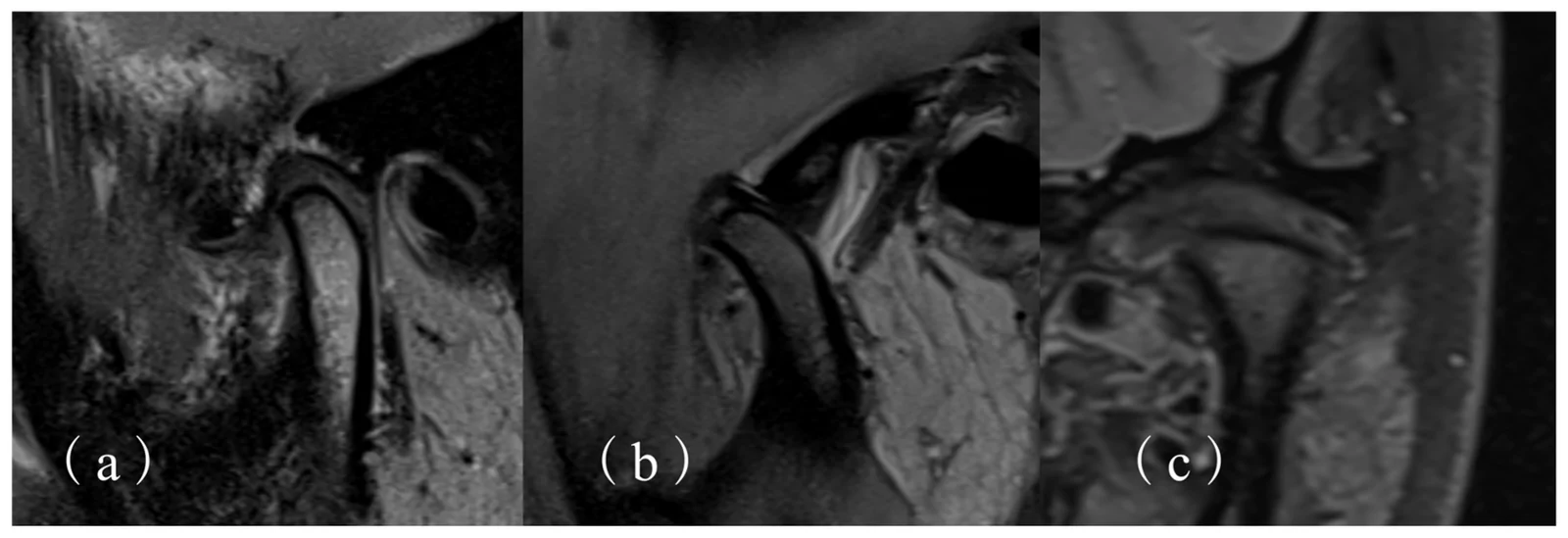
Cropped MRI images for TMJDJD classification: (**a**) sagittal view of a closed-mouth MRI image; (**b**) sagittal view of an open-mouth MRI image; (**c**) coronal view of an MRI image.

Raw MRI data were accessed and reviewed through the hospital’s imaging system. An orthodontist with five years of clinical experience used Snipaste (version 2.11.3, Shanghai, China) software to crop each image, centering on the condyle. The cropped sagittal images were resized to 512 × 512 pixels, and the coronal images were resized to 256 × 256 pixels (Figure 1). During the cropping process, the cropping frame was fixed to ensure that the condyle remained consistently positioned at the image center, thereby maintaining spatial continuity and image stability while preserving overall image quality. The cropped images encompassed key anatomical structures of the TMJ, including the condyle, glenoid fossa, articular tubercle, and articular disc. After verifying and comparing all models using the same training dataset, the cropped images were stored in PNG format, yielding 1769 sagittal and 1448 coronal images. To ensure uniformity across all images and enhance model convergence during training, the cropped MRI images were linearly normalized using AIhis (version 4.7, Fuzhou, China) software. Intensity normalization was performed independently for each image without using information from other samples.

### 2.4. Condyle Annotation

**Figure 2:**
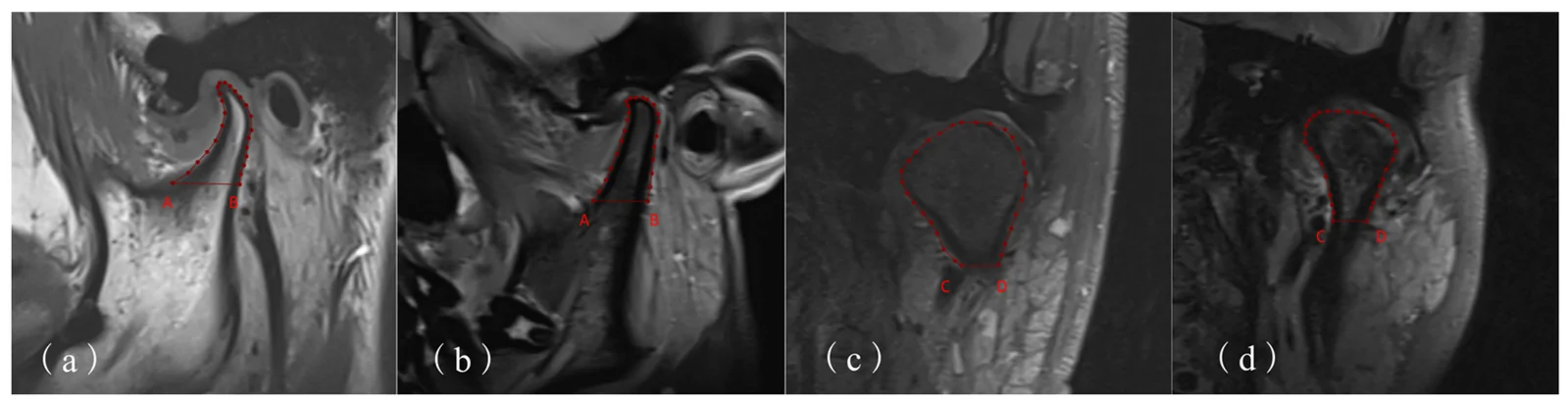
Annotation of the mandibular condyle on MRI images: (**a**,**c**) annotation method when the sigmoid notch is visible; (**b**,**d**) annotation method when the sigmoid notch is not visible.

The standardized images were imported into Labelme (version 7.0.4, MIT CSAIL) software for manual annotation. Two orthodontists, each with over 10 years of clinical experience, independently contoured the edges of the condyle manually. If there was any ambiguity in the annotation, it was adjudicated by a radiologist with over 10 years of experience. The annotation procedure followed the steps below:

For sagittal images, the lowest point of the sigmoid notch was designated as point A. From point A, a horizontal line parallel to the ground was drawn posteriorly to intersect the posterior condylar edge; this intersection was labeled point B. The line connecting points A and B defined the lower boundary of the condyle. The actual condylar contour was then traced along the outer edge from point A to point B (Figure 2a). When no sigmoid notch was visible, the lowest point on the anterior edge of the condyle was designated as point A, and the subsequent steps were performed as described above (Figure 2b).

For coronal images, if the condyle was visible but the mandibular ramus was not, the lowest point on the medial edge of the condyle was marked as point C. A horizontal line was drawn from point C until it intersected the lateral edge of the condyle at point D. The straight line connecting points C and D was defined as the lower boundary of the condyle. The outer contour of the condyle was then traced sequentially between points C and D (Figure 2c). When both the condyle and mandibular ramus were visible, the deepest concave point in the transition from the condyle to the ramus was defined as point C, and annotation was performed in the same manner (Figure 2d). All annotations were subsequently reviewed by another senior orthodontist to ensure consistency and accuracy.

### 2.5. MRI Image Grouping

The reference label for each MRI slice was derived from the CBCT-based diagnosis of the corresponding temporomandibular joint, as described above. No MRI findings were used to establish the reference diagnosis. The sagittal and coronal images were categorized into the following groups (Table 1). The sequence group was classified according to the MRI sequence (PD or T1) of all sagittal images, while the closed-mouth group included only sagittal images acquired in the closed-mouth position.

- Sagittal (Sg) group: This group was divided into the temporomandibular joint degenerative joint disease (Sg-TMJDJD) and non-degenerative (Sg-UTMJDJD) groups.
- Sequence group: All sagittal images were initially divided into the proton density-weighted (PD) and T1-weighted (T1) groups based on the MRI sequences. Depending on the presence or absence of TMJDJD, each sequence group was further divided into PD-TMJDJD, PD-UTMJDJD, T1-TMJDJD, and T1-UTMJDJD.
- Closed-mouth (Close) group: Excluding open-mouth images, the remaining closed-mouth images were divided into the closed-TMJDJD and closed-UTMJDJD groups according to the presence of TMJDJD.
- Coronal (Cor) group: The coronal images were classified into the temporomandibular joint degenerative joint disease group (Cor-TMJDJD) and non-degenerative (Cor-UTMJDJD) groups.

### 2.6. Dataset Division

The dataset was divided into training, validation, and testing sets according to an 8:1:1 ratio. Data splitting was performed at the patient level to avoid data leakage. Therefore, all images from the same patient, including both TMJs, imaging sequences, and mouth positions, were assigned exclusively to one subset. The numbers of patients, joints, and MRI images in each subset are summarized in Supplementary Table S1. The training set was used for model training, the validation set was used for hyperparameter tuning and model selection, and the testing set was held out for final performance evaluation. Data augmentation was applied only to the training set after data splitting. No augmented images were included in the validation or testing sets.

### 2.7. Classification Model Construction

**Figure 3:**
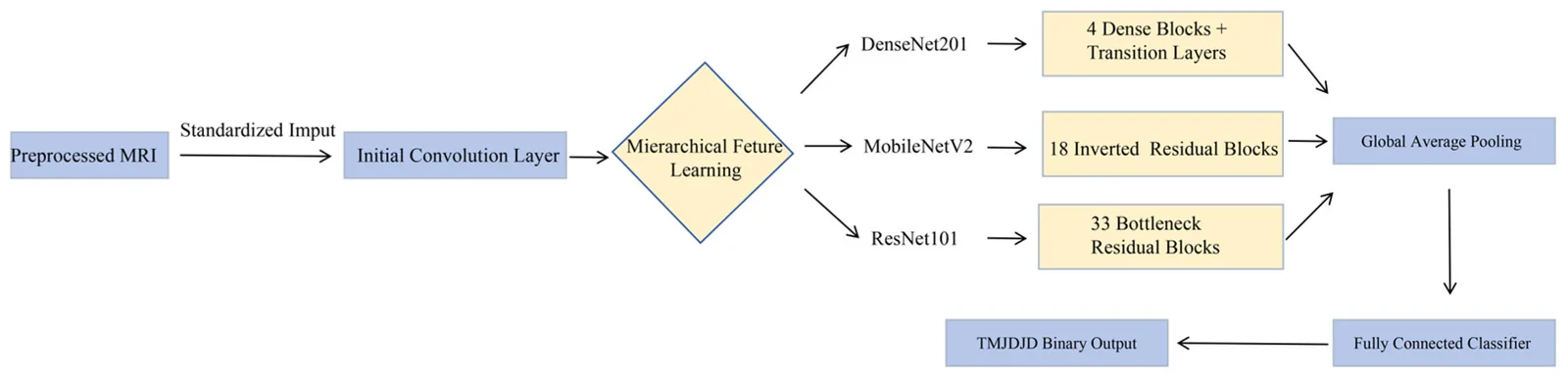
Schematic diagram of the unified deep learning pipeline for TMJDJD classification.

As described above, three representative DL architectures—ResNet101, DenseNet201, and MobileNetV2—were employed for image classification. To ensure consistency in model evaluation, identical hyperparameter settings were applied to all models. Three deep learning architectures, including DenseNet201, ResNet101, and MobileNetV2, were developed for binary classification of TMJDJD. All models were implemented using PyTorch (version 1.10.0) with ImageNet-pretrained weights and fine-tuned on the TMJDJD MRI dataset. The models were optimized using stochastic gradient descent (SGD) with an initial learning rate of 0.0025, momentum of 0.9, and weight decay of 0.0001. A class-weighted cross-entropy loss was used to alleviate the influence of class imbalance. The class weights were calculated based only on the training set distribution, resulting in weights of 0.8 and 2.0 for the non-degenerative and degenerative classes, respectively. The learning rate was adjusted using a MultiStepLR scheduler, and models were trained for 500 epochs with a batch size of 64. Data augmentation, including random resized cropping and horizontal flipping, was applied only to the training set. A fixed random seed (429457624) was used to ensure reproducibility. Early stopping was monitored based on validation performance with a patience of 100 epochs; however, the early stopping criterion was not triggered during training. Model selection was based on validation-set performance, and final evaluation was conducted on the independent testing set. Training was performed on an NVIDIA GeForce RTX 3090 GPU using CUDA 11.3. Figure 3 also offers a detailed introduction to the basic framework of each model.

DenseNet201: The preprocessed MRI images were first passed through a 7 × 7 convolutional layer followed by a 3 × 3 max-pooling layer to extract initial features. The resulting feature maps were then processed sequentially through four dense blocks, interleaved with three transition layers. These dense blocks contained 6, 12, 32, and 32 bottleneck units, respectively. Each transition layer, positioned between consecutive dense blocks, performed downsampling via a 1 × 1 convolution followed by 2 × 2 average pooling. After the final dense block, the feature map comprised 1664 channels and was fed into a classification head consisting of a 7 × 7 global average pooling layer and a fully connected layer for binary classification of TMJDJD presence in the condyle.

MobileNetV2: The MRI images were initially processed by a 3 × 3 convolutional layer for preliminary feature extraction. The resulting feature maps were subsequently passed through 18 inverted residual blocks, each consisting of a 1 × 1 expansion convolution, a 3 × 3 depthwise separable convolution, and, in selected blocks, a shortcut connection. After these layers, the feature representation contained 1280 channels at an 8 × 8 spatial resolution. A global average pooling layer followed by a fully connected layer generated the final classification output.

ResNet101: The preprocessed MRI images were first passed through a 7 × 7 convolutional layer followed by a 3 × 3 max-pooling layer, producing feature maps with dimensions of 64 × 64 × 64. These feature maps were then passed into a ResNet101 backbone composed of 33 bottleneck residual blocks. Each bottleneck block consisted of three convolutional layers (1 × 1 reduction → 3 × 3 convolution → 1 × 1 expansion) with a residual shortcut connection. After the residual blocks, the feature representation contained 2048 channels. A global average pooling layer followed by a fully connected layer generated the final binary classification output, indicating the presence or absence of TMJDJD in the condyle.

**Figure 4:**
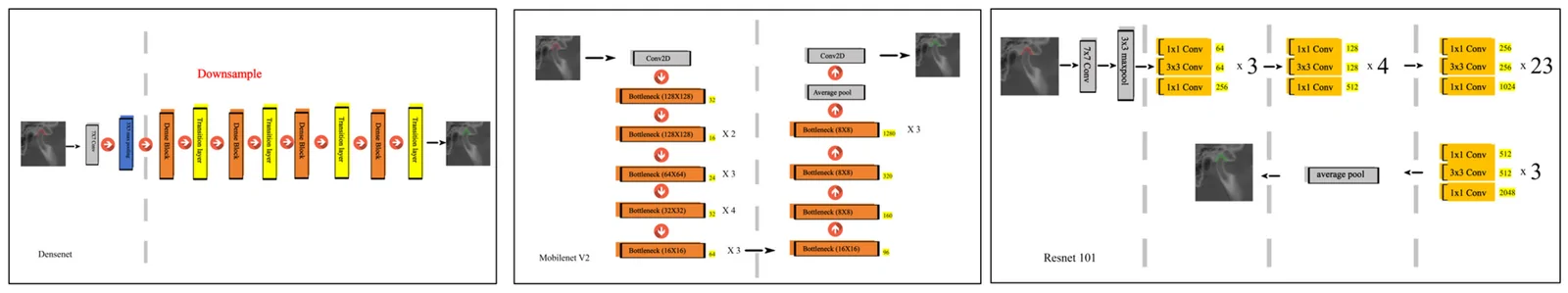
Framework and computational workflow of deep learning models. From left to right: DenseNet201 variant, MobileNetV2, and ResNet101.

**Figure 5:**
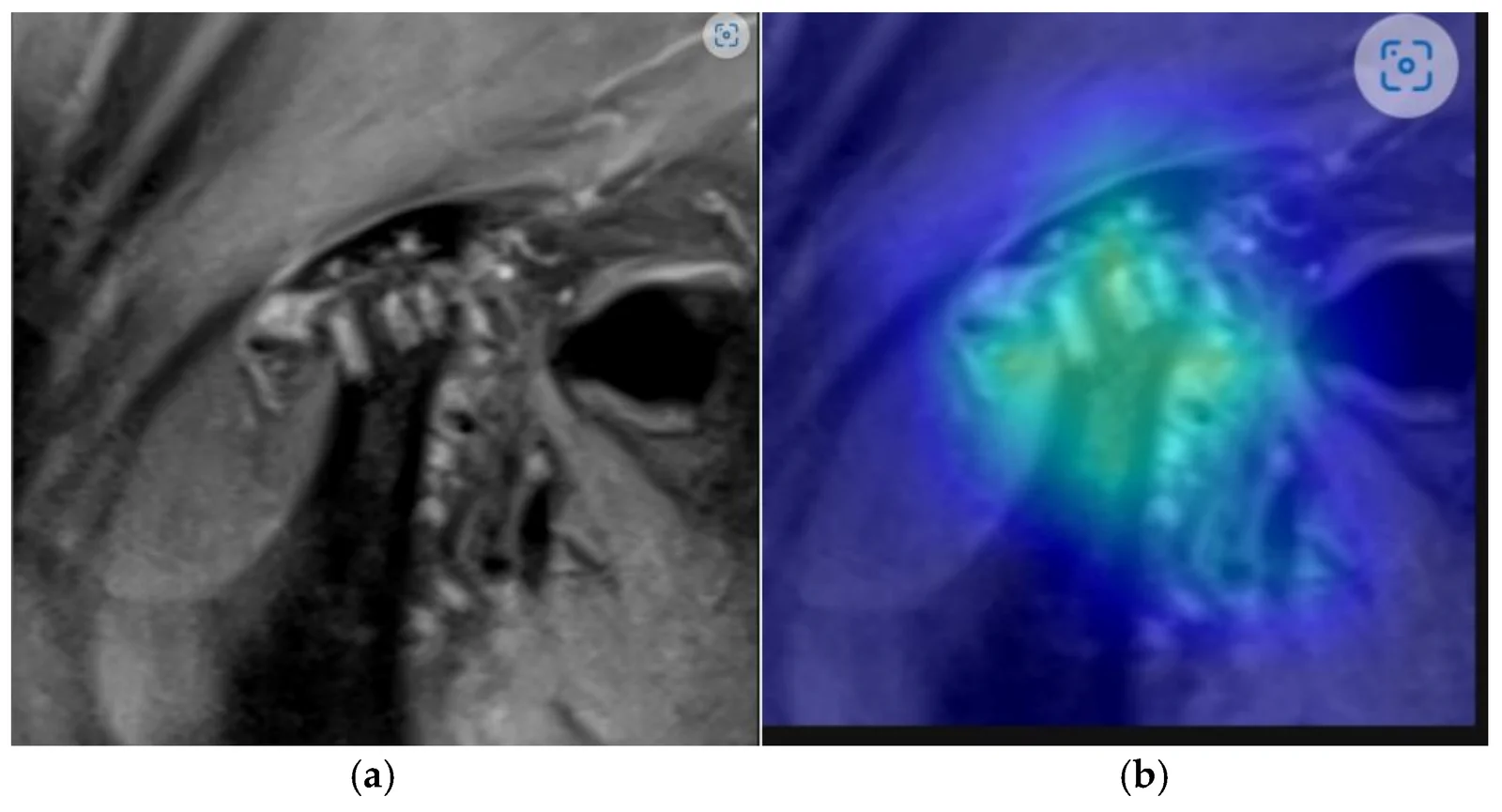
Visualization of model interpretability using Grad-CAM: (**a**) original MRI image; (**b**) Grad-CAM heatmap highlighting regions contributing most to the model’s prediction.

The overall framework and computational workflow of the three models are illustrated in Figure 4. Each model takes preprocessed images as input and outputs classified results. The heatmaps generated using Gradient-weighted Class Activation Mapping (Grad-CAM) demonstrated that the key regions identified by all three models were predominantly located in the condylar area (Figure 5).

### 2.8. Model Performance Evaluation

The classification performance of the models was evaluated using several quantitative metrics, including accuracy, recall, F1 score, Matthews correlation coefficient (MCC), specificity, false negative rate (FNR), and computational time. The predicted probabilities were converted into binary labels using a default decision threshold of 0.5, and no additional threshold optimization was performed. Receiver operating characteristic (ROC) curves were generated using predicted probabilities from the held-out testing set, and the area under the curve (AUC) values were calculated for model evaluation. Because model performance was evaluated on a held-out testing set, 95% confidence intervals for the main performance metrics were estimated using bootstrap resampling at the patient level. The high-dimensional image features extracted before the final classification layer of each trained model were visualized using t-distributed Stochastic Neighbor Embedding (t-SNE) for feature distribution analysis. Additionally, t-distributed Stochastic Neighbor Embedding (t-SNE) visualizations, along with accuracy and loss curves, were used to assess the practical applicability and training stability of the selected model.

### 2.9. Human Expert Classification

A blinded image-level reader comparison was performed to provide a human benchmark under the same constrained image-classification setting. One hundred MRI image samples were randomly selected from the held-out testing set of the Sg group and another 100 samples from the held-out testing set of the Cor group. The selected samples were balanced for the two reference categories according to the CBCT-based diagnosis.

Two senior clinical experts, each with more than 20 years of experience, independently reviewed the selected MRI images. One expert specialized in oral and maxillofacial surgery, and the other specialized in oral radiology. Neither expert had participated in the previous image annotation or model development process. During the reader experiment, the experts were blinded to the CBCT-based reference diagnosis, model predictions, and each other’s classifications. They classified each MRI image as TMJDJD or non-degenerative based only on the available MRI image and their professional judgment.

The accuracy, precision, recall, F1 score, specificity, FNR, and MCC of each expert were calculated using the CBCT-based diagnosis as the reference standard. This reader experiment was designed as an image-level comparison and was not intended to represent a full clinical diagnostic assessment based on a complete MRI series and clinical information.

## 3. Results

The CBCT-based reference diagnosis was established at the joint level for all 208 temporomandibular joints. The interobserver agreement between the two initial readers was assessed at the joint level and yielded a Cohen’s kappa value of 0.81, indicating almost perfect agreement. After consensus, the final joint-level labels were used as the reference standard for model training and evaluation. Among the 208 temporomandibular joints, the prevalence of individual CBCT-based osseous findings was as follows: subchondral cyst (*n* = 11), erosion (*n* = 25), diffuse sclerosis (*n* = 18), and osteophyte (*n* = 28). Since a single joint could present with multiple osseous alterations, these categories were not mutually exclusive.

After training the classification models, confusion matrices were generated for each deep learning model across different MRI image groups and for the human expert evaluations (Table 2). Based on these confusion matrices, performance metrics including accuracy, precision, recall, F1 score, MCC, specificity, and false-negative rate were calculated for the deep learning models (Table 3). The performance comparison between the best-performing DenseNet201 model and human experts is presented in Table 4.

### 3.1. Model Performance Across Image Groups

The classification performance of the models—ResNet101, DenseNet201, and MobileNetV2—was evaluated across different image groups, as summarized below.

Sg Group: DenseNet201 achieved the best performance in this group, with an accuracy of 80.11%, precision of 80.26%, and recall of 75.31%. Its MCC of 0.60 indicates moderate-to-strong predictive capability. However, the FNR of 24.69% suggests that it still falls short of full clinical acceptability. In contrast, both ResNet101 and MobileNetV2 performed poorly. ResNet101 exhibited an MCC of 0.01, indicating performance close to random classification.

Close Group: DenseNet201’s performance decreased noticeably in this group, with an accuracy of 64.12%. Precision, recall, and F1 score all declined to approximately 60%, accompanied by an MCC of 0.28. ResNet101 again demonstrated the weakest performance, with an accuracy of 55.73%, precision of 33.33%, F1 score of 6.45%, and a negative MCC value (−0.04), suggesting near-random classification. MobileNetV2 performed between DenseNet201 and ResNet101, but its results remained suboptimal.

PD Group: In the PD group, DenseNet201 and MobileNetV2 showed comparable performance, with accuracy, recall, and F1 scores around 65%, and an MCC value of 0.31. ResNet101 continued to exhibit low accuracy, precision, specificity, and MCC values, confirming its limited classification capability.

T1 Group: ResNet101 demonstrated advantages in recall and FNR; however, its accuracy, precision, specificity, and MCC were notably low, limiting its practical applicability. DenseNet201 maintained a reasonable balance among accuracy, precision, and MCC, although its recall remained low at 36.84%. MobileNetV2 achieved the highest accuracy and precision in this group, but its high FNR and low recall similarly restricted its clinical usefulness.

Cor Group: Although ResNet101 achieved relatively high accuracy (81.25%) and specificity (86.89%), its precision (40.74%), recall (50.00%), and F1 score (44.90%) were low. This model neither identified positive cases with sufficient precision nor captured the complete set of target instances, suggesting possible classification errors when used for diagnostic purposes. DenseNet201 performed robustly overall, with an accuracy of 82.64% and precision of 79.31%; however, the recall was only 54.76%, indicating that the model failed to correctly identify a substantial portion of positive cases. MobileNetV2 achieved an accuracy of 79.86%, but its precision, recall, and F1 score all remained around 45%, reflecting poor discriminatory ability and a considerable risk of misdiagnosis.

Some subgroup results should be interpreted cautiously because of limited sample sizes. For example, the T1 MobileNetV2 subgroup contained only one true-positive prediction, resulting in an apparently high precision but low recall, indicating unstable performance estimates.

**Figure 6:**
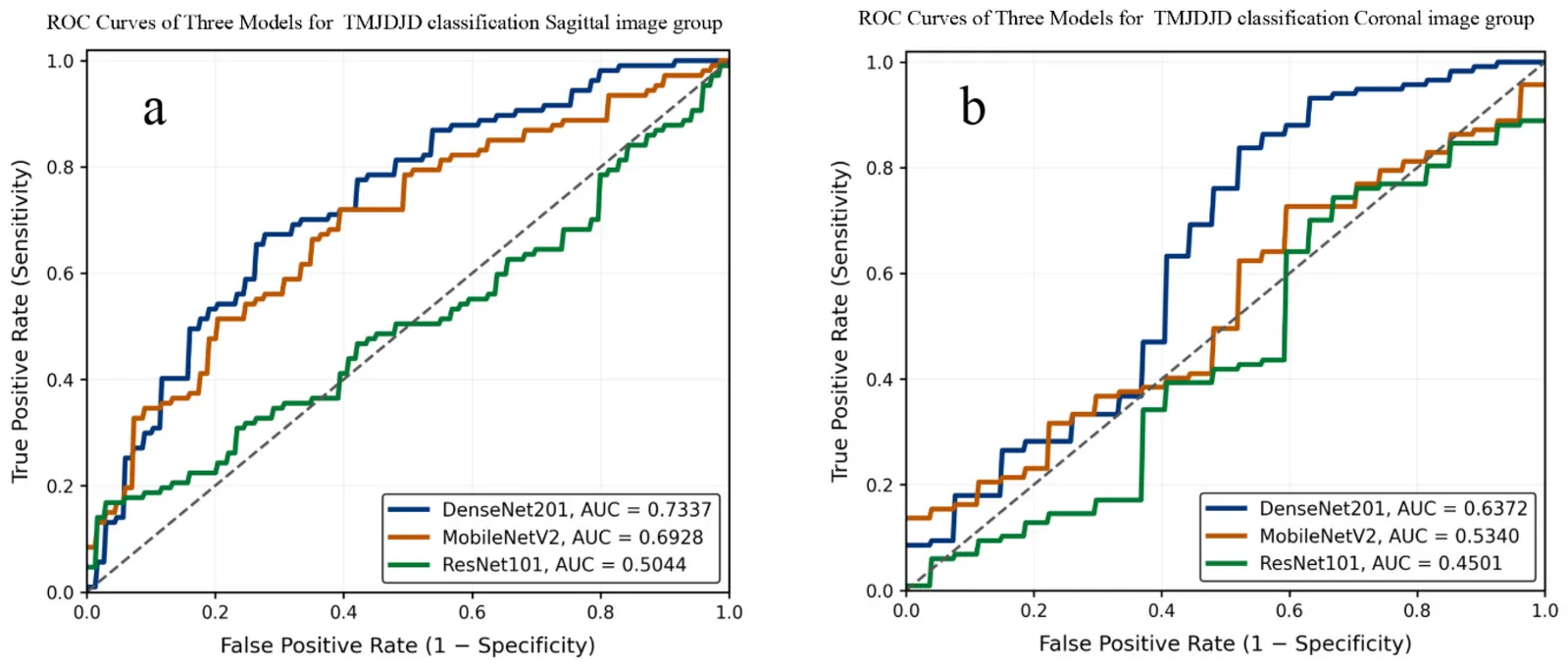
ROC curves of three deep learning models for MRI-based TMJDJD classification. (**a**) ROC curves for the sagittal image group. (**b**) ROC curves for the coronal image group. The AUC values are shown in the legend.

ROC analysis further supported the performance differences among the three models (Figure 6). DenseNet201 achieved the highest AUC in both sagittal and coronal image groups, while ResNet101 showed limited discriminative ability.

### 3.2. Threshold Analysis of DenseNet201 Model

To further investigate the influence of different decision thresholds on model performance, an additional threshold analysis was performed for the best-performing DenseNet201 model in the Sg group. As shown in Supplementary Table S2, varying the classification threshold resulted in a sensitivity–specificity trade-off. Compared with the default threshold of 0.5, lowering the threshold to 0.3 increased recall from 75.31% to 86.45% and reduced the false-negative rate from 24.69% to 13.55%, although specificity decreased from 84.21% to 54.33%. These findings indicate that threshold selection may influence the balance between missed cases and false-positive findings.

### 3.3. Image-Level Reader Comparison

**Figure 7:**
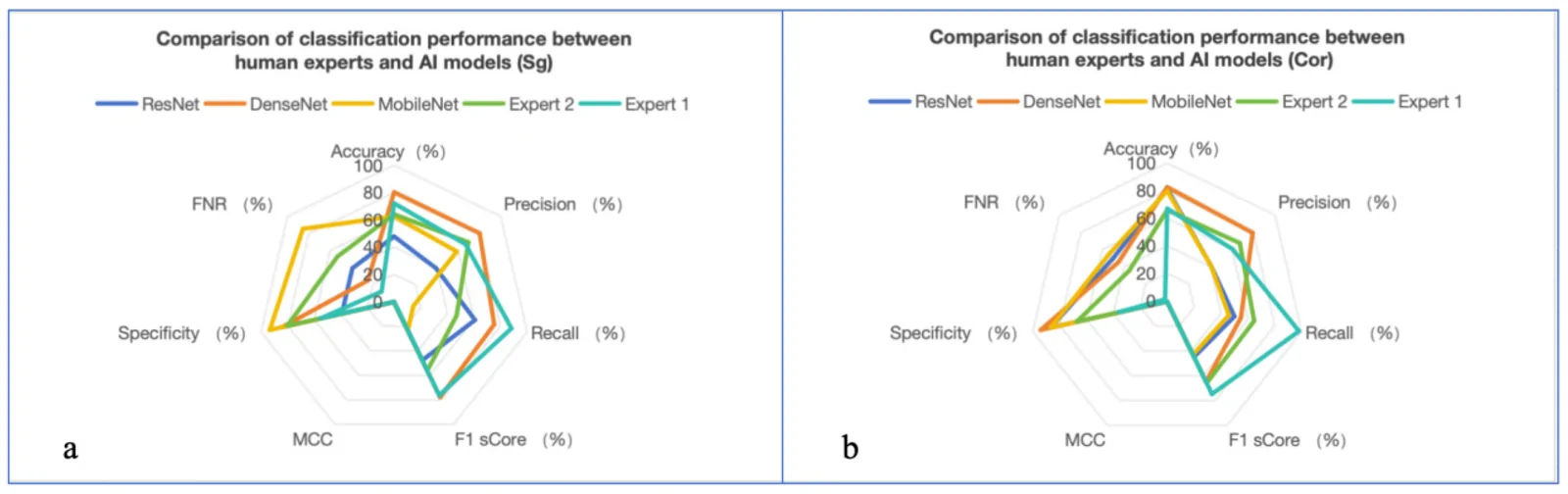
Radar chart comparing classification performance of models and human experts: (**a**) results based on sagittal images; (**b**) results based on coronal images.

The experimental findings indicated that grouping sagittal images did not improve model performance. Therefore, only the Sg group was selected as the comparison set to evaluate the classification performance of the AI models against that of human experts on sagittal images. In the constrained image-level reader comparison of sagittal images, DenseNet201 achieved higher accuracy, precision, specificity, and MCC than the two human readers. However, Expert 1 showed higher recall and a lower FNR than DenseNet201, indicating greater sensitivity to positive cases. Therefore, the reader comparison should be interpreted as evidence of complementary image-classification performance rather than superiority over clinical experts. A radar chart visually compares the performance of the classification models and human experts (Figure 7a).

In the Cor group, DenseNet201 showed higher accuracy, precision, specificity, and MCC than the human readers in this image-level task. Nevertheless, its recall remained lower than that of Expert 1, indicating that the model missed more positive cases. These findings suggest that DenseNet201 may provide useful complementary information, but its performance does not support the replacement of expert clinical assessment. A radar chart visually presents the overall performance differences between the classification models and the human experts (Figure 7b).

### 3.4. Feature Extraction Capability of Models

**Figure 8:**
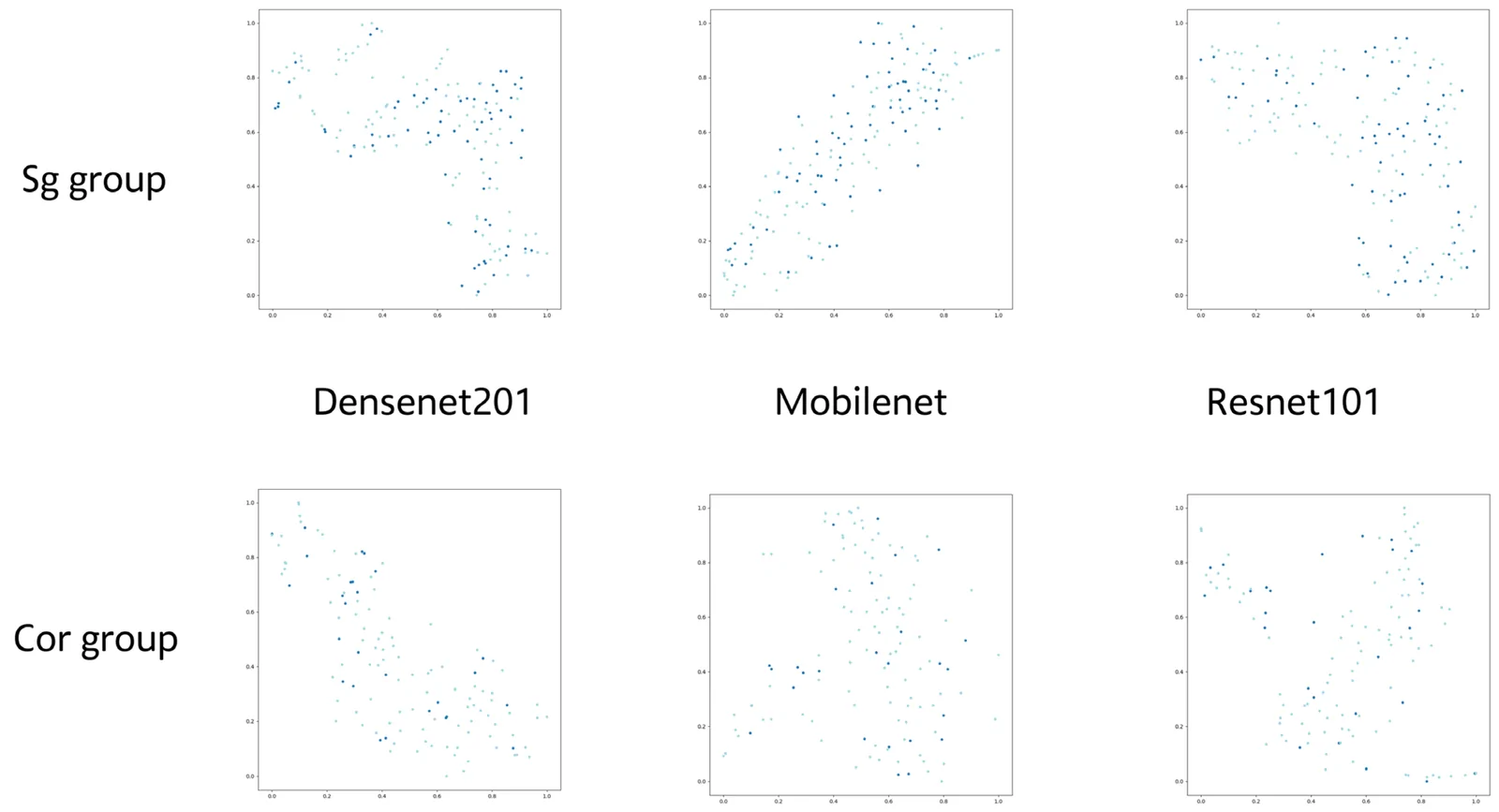
t-SNE visualization of feature distributions in the Sg and Cor groups. The features were extracted before the final classification layer of each trained model. Dark-blue and light-blue points represent TMJDJD and non-degenerative samples, respectively.

To intuitively assess the feature extraction capability of the models, t-SNE was used for dimensionality reduction (Figure 8).

In the Sg group, the t-SNE visualization of the DenseNet201 model demonstrated clearer separation between the two classes. The dark-blue points were predominantly concentrated in the lower-right region of the plot, whereas the light-blue points occupied the upper-left region, indicating DenseNet’s strong feature extraction capacity. However, intra-class cohesion remained relatively weak, as neither the dark-blue nor light-blue points formed distinct clusters—suggesting that feature representation could still be improved. In contrast, MobileNetV2 showed moderate performance: its dark-blue and light-blue points were distributed roughly along the diagonal, displaying moderate disorder and only weak-to-moderate class separation. ResNet101 performed poorly in the Sg group, exhibiting substantial inter-class mixing with indistinct boundaries.

In the Cor group, the t-SNE visualization of DenseNet201 revealed dark-blue and light-blue points distributed mainly in the central region of the diagram. Although the points were somewhat scattered, the partial class separation remained observable, yet intra-class cohesion was again weak, suggesting room for further optimization of feature extraction. Similarly, both MobileNetV2 and ResNet101 showed limited performance, as their t-SNE plots exhibited almost no inter-class separation and weak internal cohesion within the data distribution.

**Figure 9:**
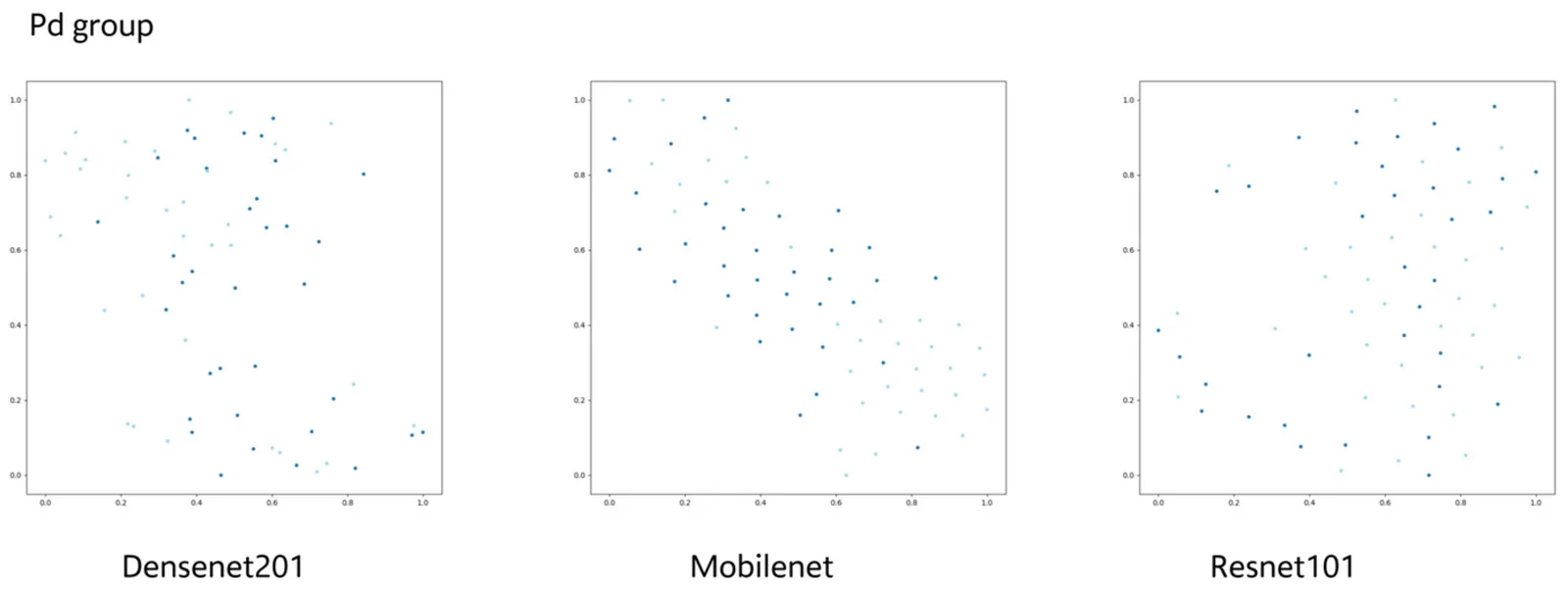
t-SNE visualization of feature distributions in the PD group.

The remaining three groups exhibited poor clustering performance in the t-SNE visualizations across all three models. For instance, in the PD group (Figure 9), the feature distributions were highly intermixed, with weak separation between classes. Even when the density of the mixed distribution appeared low, the spatial arrangement of the data points still reflected random dispersion rather than any meaningful class grouping.

### 3.5. Training Process of Models

**Figure 10:**
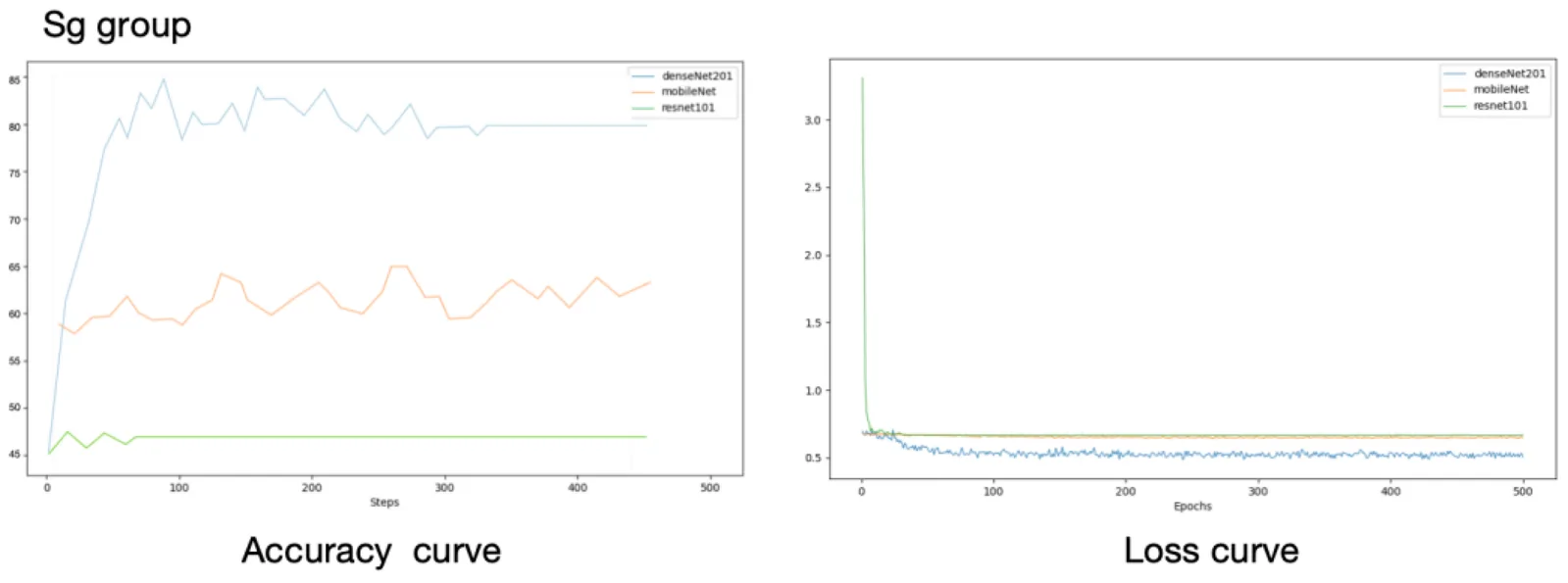
Accuracy and loss curves for the three models in the Sg group.

Figure 10 presents the accuracy and loss curves of the three models in the Sg group, illustrating their performance during training. The loss curves for all models exhibited smooth, rapid, and stable convergence, indicating that each network effectively minimized error over successive epochs. However, the accuracy curves revealed more distinct characteristics. ResNet101 showed minimal fluctuation from the early training stages and eventually stabilized around 47%. DenseNet201’s accuracy fluctuated between 75% and 85% in the early stages before achieving excellent stability. MobileNetV2 demonstrated the greatest variation among the three models, with accuracy fluctuating between 55% and 65%.

**Figure 11:**
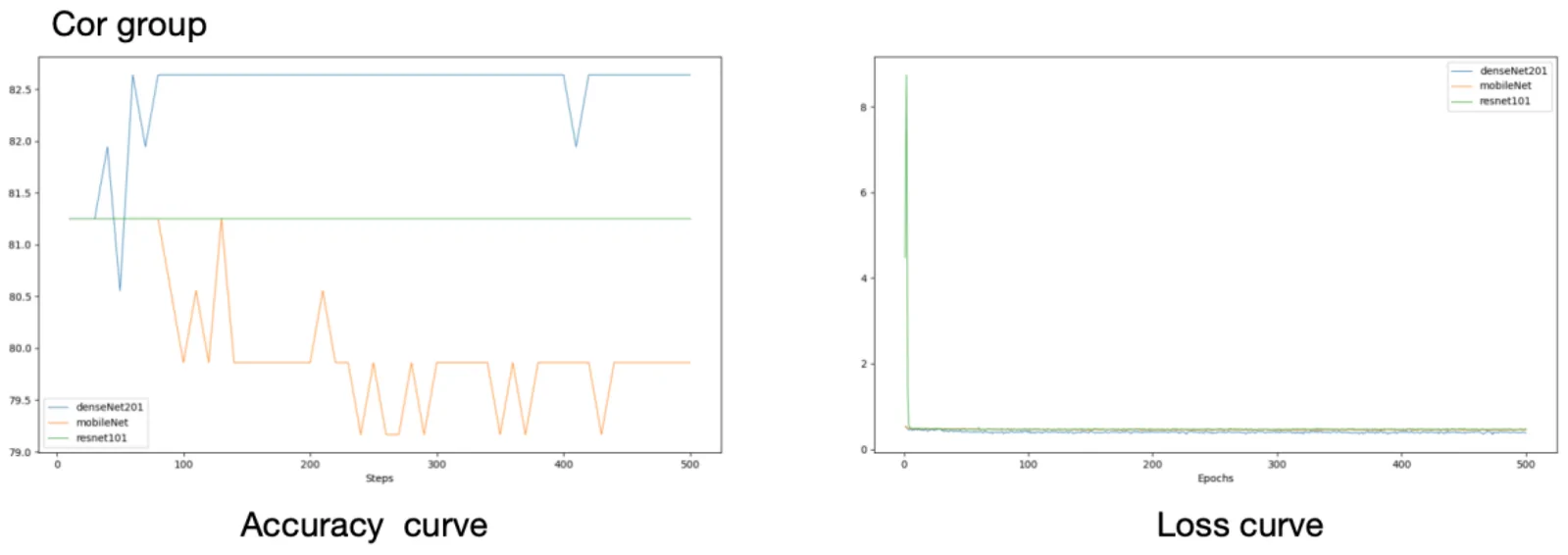
Accuracy and loss curves for the three models in the Cor group.

Figure 11 depicts the accuracy and loss curves of the three models in the Cor group, reflecting their training performance. DenseNet201 outperformed the other models, maintaining accuracy consistently above 80% and remaining stable throughout the training process, with only minor fluctuations near epoch 400. Its loss curve also showed rapid and stable convergence, reflecting strong classification capability and robust performance. MobileNetV2 exhibited lower accuracy and pronounced instability, indicating slower convergence on this dataset. Compared with DenseNet201 and MobileNetV2, ResNet101 achieved lower accuracy but displayed relatively stable operation, with its loss steadily decreasing. This pattern reflects ResNet101’s limited classification capability despite its stable training dynamics.

## 4. Discussion

MRI is widely used for soft tissue imaging and serves as a key diagnostic tool for TMD [10]. However, MRI is relatively insensitive to bone because it detects signal changes based on the movement of water molecules in a magnetic field [11]. Given the low water content of osseous tissue, MRI provides limited resolution in bone imaging. Consequently, compared with CT or CBCT, MRI has restricted utility for detailed bone assessment and lesion detection [12]. For this reason, clinicians often combine MRI and CBCT to evaluate both the soft and hard tissues of the TMJ in patients with TMD [13]. This dual-imaging strategy, however, increases examination time, financial burden, and healthcare resource utilization. If bone lesions could be accurately predicted directly from MRI scans, the dependence on CBCT could be substantially reduced, thereby minimizing radiation exposure for patients.

In this study, sagittal MRI images were categorized into four subgroups: Sg, closed-mouth, PD, and T1. During the annotation process, it was observed that open-mouth images occasionally displayed overlap between the condyle and the articular tubercle, which complicated accurate delineation. It was hypothesized that such overlap might lower classification accuracy; therefore, a closed-mouth group was created to exclude open-mouth images. Additionally, since T1-weighted and PD-weighted sequences display distinct contrast characteristics, these two groups were separated to evaluate the influence of imaging sequence type on classification performance.

From the analysis of Table 3 and Table 4, three principal observations were made. First, the performance of all three models in the closed-mouth group was lower than that in the Sg group. This finding indicates that excluding open-mouth images did not enhance classification performance, which contradicts the initial hypothesis. The overlap between the condyle and the articular tubercle did not appear to impede the models’ ability to extract features from complex anatomical images, as all three networks maintained acceptable classification accuracy.

Second, the performance of the PD and T1 groups was approximately similar, although both were inferior to that of the Sg group. This suggests that the PD- and T1-weighted sequences provide complementary diagnostic information and functional insights, consistent with the findings of Lee et al. [14]. The superior performance of the Sg group is likely attributable to its larger and more diverse dataset, which supports improved feature learning, mitigates overfitting, and enhances model generalization [15]. Finally, the results indicate that subdividing the dataset into smaller groups did not improve model accuracy. On the contrary, the reduced sample size following subdivision tended to diminish overall model performance. Therefore, maintaining adequate sample size and data diversity is essential for optimizing model performance.

Third, the analysis of the t-SNE visualizations for the sagittal image groups revealed that a model’s classification performance is not necessarily strongly correlated with its feature extraction capability. For instance, in the Sg and closed-mouth groups, DenseNet201 achieved the best classification performance, which corresponded to the greatest class separation observed in the t-SNE plot. Similar trends were also observed for MobileNetV2 and ResNet101. However, in the PD group, although all three models demonstrated relatively strong classification performance compared with the other groups, their t-SNE plots exhibited highly intermixed and random feature distributions. The t-SNE technique intuitively represents the distribution and separation of high-dimensional features in a two-dimensional projection [16]. Thus, it primarily visualizes the local structure or neighborhood relationships of the feature space, reflecting the model’s ability to extract robust representations [17]. In contrast, classification performance measures the accuracy of label assignment. Even when the t-SNE visualization displays a mixed or poorly separated feature distribution, a model can still achieve high classification accuracy if its decision boundary or internal classifier effectively distinguishes between classes [18]. Therefore, comprehensive model evaluation should adopt a multidimensional and systematic framework rather than relying solely on single classification metrics such as accuracy. Unlike many prior studies that omitted t-SNE analysis, this study incorporated such experiments to provide a more holistic assessment of model performance.

Previous research has emphasized the limitations of MRI in detecting TMJDJD, primarily due to its relatively low sensitivity and consistency in bone imaging [19]. Image interpretation often involves subjective judgment, which can result in significant interobserver variability in diagnostic outcomes. However, with the advancement of AI technologies, new perspectives in TMJDJD imaging have emerged. For instance, as early as 2014, Iwasaki et al. employed a Bayesian Belief Network (BBN) to classify osseous changes in the temporomandibular joint using MRI, achieving a classification accuracy as high as 99.49% [20]. Although this was not the focus of the present study, it exemplifies the potential of statistical algorithms to utilize MRI data for TMJDJD classification. Similarly, Orhan et al. applied radiomic feature extraction combined with multiple classifiers to evaluate both osseous alterations and disc displacement in the TMJ. They reported that the K-nearest neighbor (KNN) algorithm achieved the best performance, with area under the curve (AUC) values of 0.77 and 0.74, respectively, on the validation dataset [21]. Nevertheless, both the BBN and KNN methods represent traditional machine learning approaches rather than modern DL frameworks. Recently, Nozawa et al. applied a DL approach to PD–weighted MRI and demonstrated high diagnostic accuracy for TMJDJD, reporting that the AUC achieved by the ResNet101 model was 0.91 [22]. Compared with human observers, their AI-based method achieved or closely approached expert-level classification performance. The reported Cohen’s kappa value was 0.95, notably higher than the kappa values obtained by clinicians in the present study. However, careful comparison of the methodologies reveals key differences in both experimental design and evaluation criteria. Nozawa et al. used 200 MRI images with a resolution of 128 × 128 pixels. Although their study reported high diagnostic performance, the relatively limited number of independent samples may affect the assessment of model generalizability, as also acknowledged by the authors. The authors acknowledged this limitation in their study. In contrast, the present study utilized 1769 sagittal MRI images along with 1448 coronal images. Although the dataset contained thousands of MRI slices, these images were derived from only 104 patients and 208 temporomandibular joints. The number of independent cases available for model evaluation remained limited, and the generalizability of the proposed model requires further validation using larger multicenter datasets. Therefore, these results provide additional evidence regarding the feasibility of MRI-based TMJDJD classification, while further multicenter validation is still required.

The results of the Sg group using the DenseNet201 model represented the best-performing configuration in this study, achieving an accuracy of 80.11% and a recall of 75.31%. ROC analysis further supported the superior discriminative performance of sagittal MRI compared with coronal MRI. DenseNet201 achieved a higher AUC in the sagittal group than in the coronal group, suggesting that sagittal MRI may provide more informative features for TMJDJD classification. It should be noted that the coronal group exhibited a substantial class imbalance between degenerative and non-degenerative images (271 vs. 1177 images). Therefore, the relatively high accuracy observed in this subgroup should be interpreted cautiously, as accuracy alone may overestimate model performance under imbalanced conditions. In this context, balanced metrics such as F1 score (64.68%) and MCC (0.42) provide a more informative evaluation of model performance. These findings indicate that the model can serve as a preliminary assessment of TMJDJD. However, for a screening-oriented application, sensitivity is particularly important because missed TMJDJD cases may delay further evaluation. Although DenseNet201 achieved the best overall performance among the evaluated deep learning models, its current recall and false-negative rate indicate that it should be considered an auxiliary decision-support tool rather than a replacement for CBCT. Additional threshold analysis further demonstrated a sensitivity–specificity trade-off. Reducing the threshold from 0.5 to 0.3 increased recall and reduced false-negative cases but increased false-positive predictions. Therefore, an appropriate operating point should be determined according to the intended clinical application and the relative consequences of missed diagnosis versus unnecessary further examinations. Specifically, after MRI examination, if TMJDJD is suspected, clinicians could integrate the model’s predictions with their own clinical judgment to determine whether a CBCT scan is warranted. MRI is not routinely performed for all patients with temporomandibular disorders but is mainly used when soft tissue abnormalities or internal derangement are suspected. In these patients, the proposed model may assist clinicians in determining whether further CBCT examination is required. This approach may improve workflow efficiency for clinicians and radiologists while reducing unnecessary CBCT examinations. The stable training performance of DenseNet201 also supports its potential for future clinical translation and research applications.

An important consideration in medical imaging deep learning is the relationship between image-level prediction and clinically meaningful joint- or patient-level assessment. Although slice-level classification facilitates feature learning from individual images, its performance may not fully represent the disease status of an entire anatomical structure. Previous studies have emphasized the importance of aligning AI evaluation with the actual clinical decision unit [23]. Therefore, future studies should investigate joint-level prediction strategies, such as multi-slice aggregation, using larger datasets.

This study has several limitations. Firstly, although patient-level splitting and confidence interval analysis were performed to improve the reliability of performance evaluation, this study was based on a relatively limited single-center dataset. Differences in MRI scanners, acquisition protocols, and patient populations across institutions may affect model generalizability; therefore, external validation using larger multicenter datasets is required. Secondly, MRI and CBCT provide complementary information for TMJ evaluation. MRI is advantageous for assessing soft tissue structures and inflammatory changes, whereas CBCT remains superior for detecting subtle osseous alterations. Therefore, the proposed MRI-based deep learning model should be considered an auxiliary screening approach rather than a replacement for CBCT evaluation of bony degenerative changes. Finally, the current model was developed for MRI image-level classification rather than direct joint-level clinical decision-making. Although multiple slices from the same joint may provide complementary information, future studies should explore joint-level prediction strategies through slice aggregation methods and larger patient cohorts. Moreover, because joint-level CBCT reference labels were assigned to corresponding MRI slices, some slices may not contain visible degenerative features, which could introduce potential label uncertainty. Moreover, slice-level classification based on joint-level labels may introduce label noise because degenerative changes may not be uniformly distributed across all slices of the same joint.

## 5. Conclusions

This single-center exploratory study evaluated the feasibility of deep learning for MRI-based classification of TMJDJD using CBCT-based osseous findings as the reference standard. DenseNet201 achieved the best overall performance among the evaluated architectures, particularly in the sagittal image group. Based on image-level classification results, the model may provide preliminary support for identifying MRI findings associated with TMJDJD and assisting decisions regarding further CBCT evaluation. Nevertheless, because the model was developed and tested on a limited single-center dataset and still showed a clinically relevant false-negative rate, it should not be used as a standalone diagnostic system or as a replacement for CBCT. Larger multicenter studies, external validation, and further optimization toward higher sensitivity are needed before clinical deployment.

## Figures and Tables

**Table 1:** Image Grouping Details.

	Group	Sg	PD	T1	Close	Cor
Category						
TMJDJD		699	289	452	562	271
UTMJDJD		1070	411	483	756	1177

Sg: Sagittal group; PD: Proton density group; T1: T1-weighted group; Close: Closed-mouth group; Cor: Coronal group; TMJDJD: Temporomandibular joint degenerative joint disease; UTMJDJD: non-degenerative temporomandibular joint disorders.

**Table 2:** Confusion Matrices of Three Models and Experts.

Group	Model	True Positive	False Positive	True Negative	False Negative
SG	DenseNet201	61	15	80	20
	MobileNetV2	10	7	100	59
	ResNet101	42	65	42	27
	Expert 1	48	14	37	1
	Expert 2	23	10	41	26
Close	DenseNet201	35	26	49	21
	MobileNetV2	17	15	60	39
	ResNet101	2	4	71	54
PD	DenseNet201	22	11	24	13
	MobileNetV2	24	13	22	11
	ResNet101	32	34	1	3
T1	DenseNet201	7	7	32	12
	MobileNetV2	1	0	39	18
	ResNet101	16	35	4	3
Cor	DenseNet201	23	6	96	19
	MobileNetV2	11	16	104	13
	ResNet101	11	16	106	11
	Expert 1	50	34	16	0
	Expert 2	23	14	36	27

**Table 3:** Performance of deep learning models across MRI image groups.

Group	Model	Accuracy (%)	Precision (%)	Recall (%)	F1 Score (%)	MCC	Specificity (%)	FNR (%)
SG	DenseNet201	80.11 (76.31–83.37)	80.26 (78.14–84.51)	75.31 (69.51–80.56)	77.72 (74.47–81.82)	0.60 (0.51–0.67)	84.21 (80.07–89.66)	24.69 (20.13–28.56)
	MobileNetV2	62.50 (54.62–68.77)	58.82 (50.06–90.11)	14.49 (10.09–26.49)	23.25 (19.33–34.79)	0.13 (0.09–0.31)	93.46 (88.26–95.99)	85.51 (77.62–90.68)
	ResNet101	47.73 (31.31–59.98)	39.25 (21.78–51.77)	60.87 (45.42–64.12)	47.73 (41.13–53.66)	0.01 (0.0–0.07)	39.25 (25.18–43.97)	39.13 (26.41–53.82)
Close	DenseNet201	64.12 (59.81–70.99)	57.38 (49.03–61.76)	62.50 (60.15–69.44)	59.85 (56.24–64.06)	0.28 (0.01–0.39)	65.33 (56.31–73.79)	37.50 (30.11–41.44)
	MobileNetV2	58.78 (50.89–65.77)	53.13 (41.93–60.08)	30.36 (21.13–43.87)	38.67 (36.05–44.01)	0.12 (0.01–0.26)	80.00 (66.51–83.78)	69.64 (66.90–73.88)
	ResNet101	55.73 (49.36–61.02)	33.33 (30.95–40.59)	3.57 (0.31–8.46)	6.45 (2.31–12.11)	−0.04 (−0.87–0.38)	94.67 (76.31–98.02)	96.43 (76.31–99.08)
PD	DenseNet201	65.71 (59.13–69.56)	66.67 (60.15–78.44)	62.86 (58.33–65.35)	64.66 (60.91–70.16)	0.31 (0.11–0.91)	68.57 (56.24–75.06)	37.14 (26.33–53.07)
	MobileNetV2	65.71 (60.97–69.95)	64.86 (61.72–77.76)	68.57 (61.29–73.73)	66.67 (56.87–83.19)	0.31 (−0.09–0.57)	62.86 (56.09–73.44)	31.43 (17.39–53.34)
	ResNet101	47.14 (31.55–64.73)	48.48 (31.66–56.65)	91.43 (76.11–93.78)	63.34 (59.17–70.87)	−0.12 (−0.3–0.37)	2.86 (1.33–4.13)	8.57 (0.31–12.32)
T1	DenseNet201	67.24 (55.31–83.11)	50.00 (45.04–53.88)	36.84 (26.98–43.09)	42.86 (40.71–48.68)	0.22 (0.03–0.83)	82.05 (76.66–85.07)	63.16 (46.31–83.66)
	MobileNetV2	68.97 (57.29–80.44)	100.00 (78.07–100.00)	5.26 (2.91–8.22)	10.00 (6.26–13.49)	0.19 (−0.19–0.22)	100.00 (93.01–100.00)	94.74 (76.31–98.15)
	ResNet101	34.48 (30.47–50.49)	31.37 (17.69–54.37)	84.21 (77.08–89.17)	45.71 (26.66–63.87)	−0.08 (−0.96–0.01)	10.26 (7.07–23.35)	15.79 (4.31–33.06)
Cor	DenseNet201	82.64 (77.15–88.58)	79.31 (76.42–82.68)	54.76 (50.56–60.78)	64.82 (59.04–70.67)	0.55 (0.43–0.7)	94.12 (89.55–95.41)	45.24 (40.98–49.28)
	MobileNetV2	79.86 (70.31–87.61)	40.74 (39.51–43.55)	45.83 (43.31–48.07)	43.12 (35.13–52.83)	0.31 (0.11–0.45)	86.67 (64.31–90.02)	54.17 (46.91–63.17)
	ResNet101	81.25 (66.81–93.37)	40.74 (32.64–49.96)	50.00 (34.94–61.01)	44.90 (36.11–57.55)	0.34 (0.02–0.65)	86.89 (80.51–90.79)	50.00 (36.75–61.53)

**Table 4:** Human expert comparison.

Group	Model	Accuracy (%)	Precision (%)	Recall (%)	F1 Score (%)	MCC	Specificity (%)	FNR (%)
SG	DenseNet201	80.11 (76.31–83.37)	80.26 (78.14–84.51)	75.31 (69.51–80.56)	77.72 (74.47–81.82)	0.60 (0.51–0.67)	84.21 (80.07–89.66)	24.69 (20.13–28.56)
	Expert 1	72.00 (60.17–85.44)	67.16 (64.28–70.75)	88.24 (70.69–90.22)	76.29 (66.68–84.49)	0.27 (0.06–0.33)	55.10 (32.05–62.97)	11.76 (9.58–33.26)
	Expert 2	64.00 (45.58–80.09)	69.70 (55.91–80.03)	46.94 (37.64–51.83)	56.12 (47.73–62.11)	0.29 (0.12–0.31)	80.39 (65.82–90.01)	53.06 (33.51–64.89)
Cor	DenseNet201	82.64 (77.15–88.58)	79.31 (76.42–82.68)	54.76 (50.56–60.78)	64.82 (59.04–70.67)	0.55 (0.43–0.7)	94.12 (89.55–95.41)	45.24 (40.98–49.28)
	Expert 1	67.00 (53.26–74.33)	60.49 (46.12–81.37)	98.00 (89.44–99.08)	74.80 (66.21–79.51)	0.29 (0.17–0.34)	36.00 (26.81–45.85)	2.00 (0.98–7.79)
	Expert 2	66.00 (45.55–79.78)	67.35 (60.91–74.29)	64.71 (55.04–77.01)	65.97 (60.96–79.71)	0.32 (0.21–0.37)	67.35 (46.01–80.51)	35.29 (25.73–48.97)

## Data Availability

Due to ethical and legal restrictions, the authors cannot publicly release patients’ MRI images or related medical data. The source code used to construct the models is available upon reasonable request from the corresponding author via the team’s GitHub repository.

## Supplementary Materials

The following supporting information can be downloaded at: https://www.mdpi.com/article/10.3390/jcm15166225/s1, Table S1: Distribution of patients, joints, and MRI images across training, validation, and testing sets; Table S2: Performance of DenseNet201 under different classification thresholds.

## Abbreviations

AI	Artificial intelligence
CT	Computed tomography
CBCT	Cone-beam computed tomography
MRI	Magnetic resonance imaging
TMJ	Temporomandibular joint
TMD	Temporomandibular joint disorder
TMJDJD	Temporomandibular joint degenerative joint disease
DL	Deep learning
FNR	False negative rate
MCC	Matthews correlation coefficient
